# Intrinsic Brain Connectivity Related to Age in Young and Middle Aged Adults

**DOI:** 10.1371/journal.pone.0044067

**Published:** 2012-09-11

**Authors:** Michelle Hampson, Fuyuze Tokoglu, Xilin Shen, Dustin Scheinost, Xenophon Papademetris, R. Todd Constable

**Affiliations:** 1 Department of Diagnostic Radiology, Yale University School of Medicine, New Haven, Connecticut, United States of America; 2 Department of Biomedical Engineering, Yale University, New Haven, Connecticut, United States of America; University Of Cambridge, United Kingdom

## Abstract

Age-related variations in resting state connectivity of the human brain were examined from young adulthood through middle age. A voxel-based network measure, degree, was used to assess age-related differences in tissue connectivity throughout the brain. Increases in connectivity with age were found in paralimbic cortical and subcortical regions. Decreases in connectivity were found in cortical regions, including visual areas and the default mode network. These findings differ from those of recent developmental studies examining earlier growth trajectories, and are consistent with known changes in cognitive function and emotional processing during mature aging. The results support and extend previous findings that relied on a priori definitions of regions of interest for their analyses. This approach of applying a voxel-based measure to examine the functional connectivity of individual tissue elements over time, without the need for a priori region of interest definitions, provides an important new tool in brain science.

## Introduction

Human aging throughout the lifespan is associated with many changes including improvements in emotion regulation [1] and declines in sensory and cognitive function across a variety of domains [2], [3], [4], [5]. Identifying the neurobiological bases of these changes is important not only for aging research, but for cognitive neuroscience more generally, as it provides clues into the neural basis of those mental functions that are changing with age.

A common approach in aging research is to contrast young and old adults, without including a middle age cohort [6], [7], [8]. However, those studies that have examined changes in brain function throughout the lifespan have typically reported gradual and continuous changes that begin in early adulthood and extend throughout middle age and senescence [3], [9]. Therefore, a complete understanding of the aging process requires the characterization of changes occurring in young and middle aged adults. Here we examine age-related differences in the intrinsic connectivity patterns of healthy adults in this age range.

Brain imaging studies of healthy human aging have reported changes in structure, activation, and/or connectivity patterns associated with aging in many different regions of the brain, including, but not limited to, fronto-striatal circuits [10] and the so-called “default mode network” [7], [8], [9]. More specifically, frontal cortices have been reported to have age-related decreases in volume [11] and resting cerebral blood flow [12], and age-related increases in task activation [13], [14]. The default mode network has been reported to have age-related decreases in task deactivation [9], grey matter volume [8], and resting state connectivity [7], [8]. Imaging studies have also implicated many other areas of the brain in aging. For example, studies have shown decreased activation in occipital regions [13], [14] and both increased activation [13] and decreased volume [15] in parietal regions. The distributed and complex nature of these changes highlights the need for network level analyses of the aging human brain.

An emerging approach for studying human brain networks involves the application of graph theory [16]. Studies of this nature, examining age-related changes in structural and functional brain networks, have reported decreases in whole-brain efficiency [6], decreases in cortical connectivity and local efficiency [17], and changes in the modularity structure of the brain of older adults [18]. However, one of the challenges inherent in applying graph theory to functional imaging data is the definition of network nodes. A variety of approaches can be used to define network nodes, including parcellations based on functional similarity metrics [19] or anatomy [20], or the definition of a specified set of regions of interest based on prior literature [21]. Unfortunately, assumptions incorporated into node definition can have tremendous impacts on the resulting conclusions. For example, if a region is defined spanning functional areas with very different patterns of temporal activity, the timecourse of activity in the region will be an average of the timecourses from the functional areas comprising it. Although each of the functional areas may have strong correlations with other brain areas, those connections are unlikely to be identified when correlating to this averaged time course. To the extent that age-related brain changes involve alterations in the spatial extent of specific functional areas, this can result in different estimates of connectivity with age that are unrelated to connectivity per se. This problem tends to be more pronounced when larger regions are used as network nodes, because the regions are more likely to span functionally disparate brain areas.

Here we adopt an approach that minimizes this problem: each voxel is defined as a network node. Although computationally expensive, this approach allows unbiased exploration of the network properties of the human brain. It has revealed that the human brain has a small-world, scale-free functional architecture [22]. Voxel-wise network analyses have been used successfully to identify hubs in the human brain [23], [24], [25], to highlight regions of the brain where network efficiency is related to cognitive function [26], to study how exercise affects brain function in older adults [27] and to investigate the impact of anesthetic agents on the brain [28]. In this work we apply this approach to investigate the changes in network connectivity patterns associated with healthy aging.

When applying graph theory to study human functional brain networks, the functional imaging data must first be translated into either a weighted or unweighted graph, after which one or more network properties of interest can be computed. Most prior studies have modeled functional human brain networks using unweighted graphs [6], [23], [26], [27], [29]. However, this approach discards potentially valuable information regarding the strength of each connection in a graph. An alternative made possible by recent developments in weighted graph theory is to maintain information regarding the strength of each existent connection and to use that information in computing network measures. Here we explored both approaches. Using an approach similar to that shown by Buckner et al. [23], we computed the unweighted network measure of degree in a voxel-wise manner and examined the relationship between this network measure and age. In this voxel based approach the intensity of each voxel reflects the number of connections that voxel has (with correlations r>0.25) to the rest of the voxels in the gray matter. As such, a high degree measure implies that that tissue element is highly connected to the rest of the brain while a low measure of degree implies fewer connections to other brain tissue. Second, we computed the weighted graph measure of vertex strength: for each node (or vertex) in the graph, this is a summary measure of the strength of all connections to that node [30]. Thus, it is an extension of the network measure of degree to a weighted graph context.

In summary, we present here an exploratory examination of age-related differences in intrinsic connectivity patterns of healthy young to middle-aged adults. Voxel-wise network measures are used, allowing an approach that is unbiased by a-priori expectations regarding regions of interest.

## Methods

### Ethics Statement

All subjects provided a written informed consent in accordance with a protocol approved by the Human Research Protection Program of Yale University.

### Subjects

**Figure 1:**
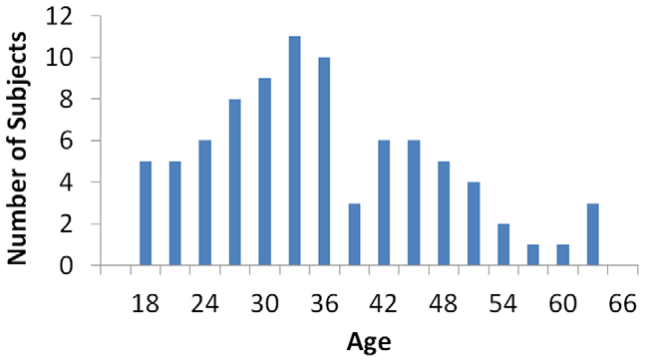
Age distribution of subjects.

Ninety-one healthy right-handed subjects aged 18 to 65 participated in the study, all of who reported no history of psychiatric or neurological illness. Of these, 3 were removed due to excessive head motion, 3 were removed due to brain abnormalities (as determined by a radiologist) and 85 were included in the analyses. The analyses included 41 females (mean age 35) and 44 males (mean age 35). Figure 1 shows the age distribution of subjects.

### Imaging Protocol

Subjects were scanned on 3T Siemens Trio scanners. Of the 85 subjects, 59 were scanned on one scanner (referred to here as Trio A) with a 12 channel head coil. The remaining 26 were scanned on a second Trio (referred to here as Trio B) with a 32 channel head coil. There was no significant difference in age for the subjects scanned on the two scanners (Trio A mean: 35.85, std: 10.74, Trio B mean: 32.27, std: 12.51).

Each session began with a localizing scan, followed by a low-resolution sagittal scan for slice alignment, and the collection of 25 6 mm thick axial-oblique T1-weighted slices aligned with the AC-PC such that the top slice was at the top of the brain. Resting state functional data was collected at the same slice locations as the T1-weighted anatomical data, using a T2*-sensitive gradient-recalled single shot echo-planar pulse sequence (TR = 1550 ms, TE = 30 ms, flip angle = 80 degrees, FOV = 220^2^ mm, 64^2^matrix). Subjects were instructed to rest with their eyes open, not to think of anything in particular and not to fall asleep. There were eight functional runs, each comprised of 240 volumes, the first six volumes were discarded to allow the signal to reach a steady-state. Finally, a high-resolution anatomical image was collected using an MPRAGE sequence (TR = 2530 ms, TE = 2.77 ms, TI = 1100 ms, flip angle = 7 degrees, resolution = 1 mm*1 mm*1 mm).

### Data Analysis

Data were analyzed as described in previous studies that computed degree on a voxel-wise basis [23], [31]. Analyses were conducted in the BioImage Suite software package (www.bioimagesuite.org) except in a few instances where modules from other programs were used. In these cases, the software that was used is noted in the text.

#### Preprocessing

All data were first adjusted for different slice acquisition times using sinc interpolation in Matlab (www.mathworks.com) and then motion corrected using the SPM5 algorithm (http://www.fil.ion.ucl.ac.uk/spm/software/spm5/). To remove the potential influence of spurious or spatially nonspecific sources of correlation, the time-courses of several variables of no interest were removed by regression. These included the six motion parameter time-courses, the average signal in the white matter, the average signal in the cerebrospinal fluid, and the whole-brain time-course. Finally, data were temporally smoothed using a Gaussian filter with a cutoff frequency of 0.1 Hz.

#### Computation of degree maps

The time course for each voxel i was used as a reference time-course and correlated with the time course of every other voxel in the grey matter of the brain, resulting in a seed map of correlations to voxel i. This map was converted to a binary map of connectivity to the seed region, by thresholding at r = 0.25, setting all connections below that threshold to zero, and setting all remaining connections to 1. The sum of all the connections in this binary map was computed to yield D_i_, the degree of connectivity of voxel i. This process was repeated for each voxel in the brain to yield a whole-brain map of the network measure degree.

For each subject, the whole brain maps of degree were then standardized to z-score maps as described in Buckner et al (2009) using the equation Z_j_ = (D_j_−μ_D_)/σ_D_ for each voxel j where μ_D_ is the mean degree across all voxels and σ_D_ is the standard deviation of degree across all voxels. This ensured that the maps of degree for all subjects had a similar scale.

To investigate whether this normalization could introduce age-related effects, we correlated each of the two parameters of the normalization (μ and σ), and the ratio between these two parameters, with age.

The threshold used to compute degree in this study was chosen to be consistent with Martuzzi et al (2010) [31] and Buckner et al (2009) [23]. Threshold-free approaches have recently been introduced, however, they tend to have less power than this threshold-based approach [31], [32] although very recent work [32] appears to overcome the sensitivity problem. To investigate sensitivity to threshold we varied it from 0.15 to 0.5 and found that the qualitative pattern of results was stable.

#### Computation of vertex strength maps

This was identical to the computation of degree maps, except when the map of connectivity to a given voxel i was thresholded, connections surviving the threshold maintained their value. Vertex strength for voxel i was computed by summing all the connections in the map. Vertex strength was also standardized using a z-score conversion as described for degree.

#### Group-level maps of changes in network measures with age

Maps from individual subjects were transformed to the coordinate space of the Colin brain [33] via a concatenation of three registrations: (i) a linear rigid transformation of the functional data to the axial-oblique anatomical data collected in the same scanning session (ii) a linear rigid transformation of the axial-oblique anatomical data to that subject's MPRAGE image and (iii) a nonlinear registration of that subject's MPRAGE image [34] to the Colin brain. All registrations were inspected visually to ensure accuracy.

Once the data were in common space, group level analyses from the 85 subjects were conducted in AFNI (http://afni.nimh.nih.gov/afni). The AFNI program 3dLME was used to compute a voxel-wise map of the main effects of age (continuous variable), controlling for the effects of scanner (categorical variable). To avoid effects related to the order of the variables in the model, marginal sum-of-squares was selected. To correct for multiple comparisons, the t-value map for age was cluster corrected (p<0.05 voxel-wise threshold and 2911 mm^3^ cluster-extent threshold to yield a p<0.05 whole-brain cluster-corrected threshold) using a Monte Carlo simulation within the AFNI program AlphaSim. Talairach coordinates for the center-of-mass of each region were then calculated using the method of Lacadie, et al. [35].

In addition, the data from the 59 subjects scanned on the Trio A were analyzed for comparison purposes. In this case, there was no scanner effect to control for, and a simple map of correlations to age was computed using BioImage Suite.

#### Addressing potential confounds related to head motion

First, estimates of total head motion were computed for each subject. Previous studies used an estimate of total displacement at a specific location in the brain [36], [37], but that approach is sensitive to the location chosen. An alternative approach was used here, where net displacements were computed for every voxel in the brain and averaged across all voxels, yielding estimates of total displacement that don't depend upon an arbitrary reference point. In computing total displacement of a voxel in a given run, two different approaches were used: (1) “frame-to-frame”: the displacement of the voxel from one frame to the next was averaged across all frames (2) “frame-to-reference”: displacement of each voxel from it's location in the reference frame to which all other frames were motion corrected was averaged across all frames. The latter measure places greater emphasis on gradual movements that shift the head from one position to another over an extended time, while the former measure emphasizes “shaky” movements where the head moves back and forth but doesn't move far from where it started.

To investigate whether head motion could be responsible for the age-related changes in connectivity, correlations between age and our two measures of head motion were computed. Neither measure was significantly correlated with age, but the frame-to-frame measure had a correlation with age that was approaching significance (p = 0.09). The frame-to-reference measure was not at all correlated (p = 0.9). Given that the frame-to-frame measure was approaching significance, further analyses were needed to ensure our results were not confounded by motion.

Two additional analyses were performed to control for head motion. First, the estimates of frame-to-frame head motion were included as a regressor in the group level analysis. The resulting map of age effects was very similar to that computed without the regressor. However, this approach is limited to removing linear effects. Therefore, a second analysis was conducted in which we threw out two runs of data for each subject. These runs were selected so as to minimize the correlation between age and head motion. After discarding these runs, we still had six runs of data for every subject and neither frame-to-frame nor frame-to-reference motion was significantly correlated with age in this subset of the data (p = 0.5 for both). The group level maps of changes in degree with age were then computed on this subset of the data in exactly the same manner as they had been computed on the full data set.

#### Secondary analyses exploring changes in seed region connectivity

The primary analyses of this study examined intrinsic connectivity changes with age. This identifies regions of the brain that change their degree of connectivity with age, however, it does not provide information about which connections are changing. To explore this, secondary seed-region analyses were conducted. We selected two key regions for follow-up seed bases analyses, the one with the greatest increase in intrinsic connectivity with age and the one with the greatest decrease in intrinsic connectivity with age. These were the medial temporal cortex and the anterior cingulate cortex, respectively.

**Figure 2:**
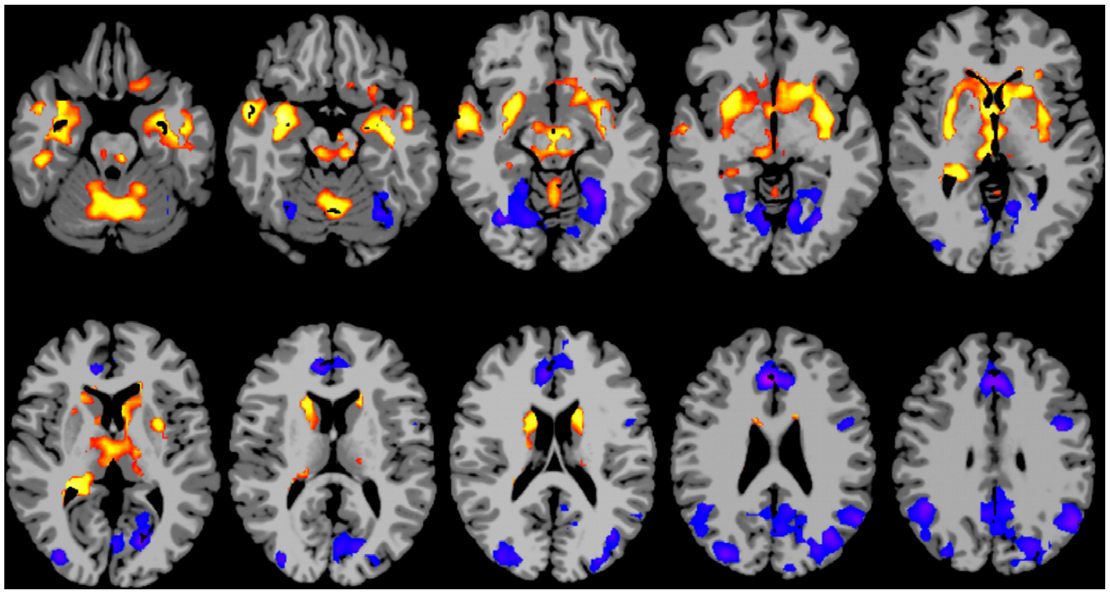
Map showing brain areas where there is a main effect of age on degree of connectivity, displayed at a whole brain corrected p<0.05 level. Red/yellow areas indicate regions where connectivity increases with age, blue/purple areas indicate regions where connectivity decreases with age. Slices are shown using radiological convention (i.e. left is on the right).

The medial temporal cortical region of interest (ROI) was defined to include all significantly positive voxels in this region (in both hemispheres) in the cluster-corrected group-level map of changes in degree with age (i.e., Figure 2). This ROI was translated back into the functional space of each subject by applying the inverse registration used to translate the functional data into MNI space. The average time-course across the region was computed and used as a reference time-course with which every other voxel in the gray matter was correlated. The resulting correlations were transformed to Gaussian variables using Fishers transform to yield a map of connectivity in that subject to the medial temporal ROI. These individual subject seed maps were then translated back into MNI space and the AFNI program 3dLME was used to compute a voxel-wise map of the main effects of age across subjects, controlling for the effects of scanner. This group map was cluster-corrected using the AFNI program AlphaSim and those areas showing significant increases in connectivity to the medial temporal ROI with age are displayed at a whole-brain corrected p<0.05 threshold.

The anterior cingulate cortical ROI was defined to include all significantly negative pixels within the anterior cingulate cortex in the cluster-corrected group-level map of changes in degree with age (i.e., Figure 2). The computation of how connectivity to this region changes with age was identical to that described for the medial temporal ROI. Those regions of the brain that had significant decreases in connectivity to the posterior cingulate cortex with age are displayed at a whole-brain corrected p<0.05 threshold.

## Results

### Changes in degree with age

The degree of connectivity was found to vary with age. Figure 2 shows a t-statistic map for the effect of age on degree at a whole-brain cluster corrected p<0.05 level. A positive relationship between degree of connectivity and age is apparent in many subcortical and paralimbic cortical regions, as summarized in Table 1. A negative relationship between degree and connectivity is apparent in the cortical regions listed in Table 2. Note these include prominent loci in default mode areas. Neither of the parameters of the normalization (μ or σ) nor the ratio between these parameters (μ/σ), were significantly correlated with age (the t and p-values of correlation to age are μ: t = 0.78, p = 0.44, σ: t = 1.23, p = 0.22, μ/σ t = −0.7,p = 0.49), indicating that these results are not due to the z-score normalization process.

**Table 1:** Regions with a positive relationship between degree of connectivity and subject age.

Region		Size (mm^3^)	x	y	z
Medial temporal cortex	R	4585	30	−7	−13
	L	4051	−28	−7	−14
Temporal pole	R	4283	40	3	−19
	L	4268	−38	0	−25
Putamen	R	5273	26	0	0
	L	6367	−27	−1	0
Caudate	R	3821	13	9	9
	L	4269	−12	9	5
Thalamus	R	2961	6	−17	4
	L	1582	−7	16	7
Tail of Caudate/Hippocampus	R	2600	24	−37	6
Parahippocampus	R	2597	28	−11	−21
	L	1450	−28	−11	−22
Inferior temporal gyrus/Fusiform	R	2408	43	−22	−21
	L	1804	−45	−16	−21
Superior Temporal Sulcus	R	2034	54	−9	−5
Orbitofrontal cortex	L	863	−18	13	−14
Hypothalamus		795	1	0	0
Subgenual cingulate		708	−2	15	−4
Globus Pallidus	R	292	16	1	0
Insula	L	240	−27	21	0
Cerebellum	R	13039	21	−57	−24
	L	11202	−14	−60	−23
Midbrain		4510	1	−23	−8
Pons/Brainstem		3673	0	−26	−29

Coordinates indicate center of mass of region in Talairach space.

**Table 2:** Regions with a negative relationship between degree of connectivity and subject age.

Region		Size (mm^3^)	x	y	z
Visual cortex	R	9820	25	−71	7
	L	17709	−21	−69	9
Medial prefrontal		10075	0	32	25
Posterior cingulate		8863	−2	−50	30
Precuneus		3164	7	−65	42
Lateral parietal	R	6481	47	−58	34
	L	9245	−42	−61	36
Precentral sulcus/middle frontal gyrus (BA 6/8/9)	L	2972	−43	6	32

Coordinates indicate center of mass of region in Talairach space.

### Changes in degree with age in those subjects scanned on the Trio A

When we limited our analyses to just those 59 subjects scanned on the Trio A, our power was significantly reduced. However, the qualitative pattern of the age effects was similar. In the Supplementary data (Figure S1) we show the map of simple correlations with age in the 59 subjects scanned on the Trio A at an uncorrected p-value of p<0.05. At this less stringent threshold, a similar pattern emerges as seen at the corrected level in the larger group.

### Changes in vertex strength with age

The map of correlations between vertex strength and age was very similar to the map of correlations between degree and age. This map is provided in the Supplementary data (Figure S2)

### Results of analyses controlling for head motion

**Figure 3:**
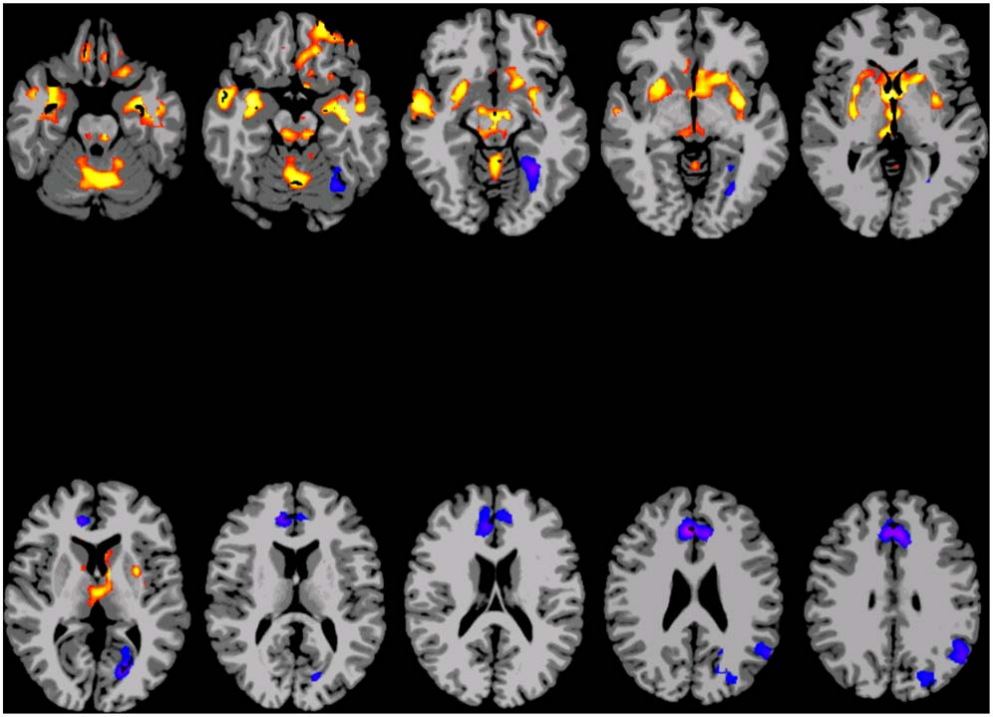
Map showing brain areas where there is a main effect of age on degree of connectivity in the subset of data where age was not correlated with head motion, displayed at a whole brain corrected p<0.05 level. Red/yellow areas indicate regions where connectivity increases with age, while blue/purple areas indicate regions where connectivity decreases with age. Slices are shown using radiological convention (i.e. left is on the right).

The inclusion of frame-to-frame head motion as a covariate in the group level analyses correlating age with degree of connectivity had very little effect on the results. The t-statistic map of age from the model including the motion covariate is provided the Supplementary data as Figure S3. It is very similar to Figure 2. As this only controls for linear effects, an analysis using a subset of data where head motion was balanced across age was also computed (six of the eight runs from each subject were included). The t-statistic map of the effect of age on degree in this subset of the data is shown at a whole-brain cluster corrected p<0.05 level in Figure 3. Although it is quite similar to Figure 2, there are some differences. Most notably, the negative correlations between age and connectivity in the posterior cingulate, the right lateral parietal, and right visual regions are not apparent in the subset of the data where motion was balanced across age (i.e., in Figure 3).

### Secondary analyses exploring changes in seed region connectivity

**Figure 4:**
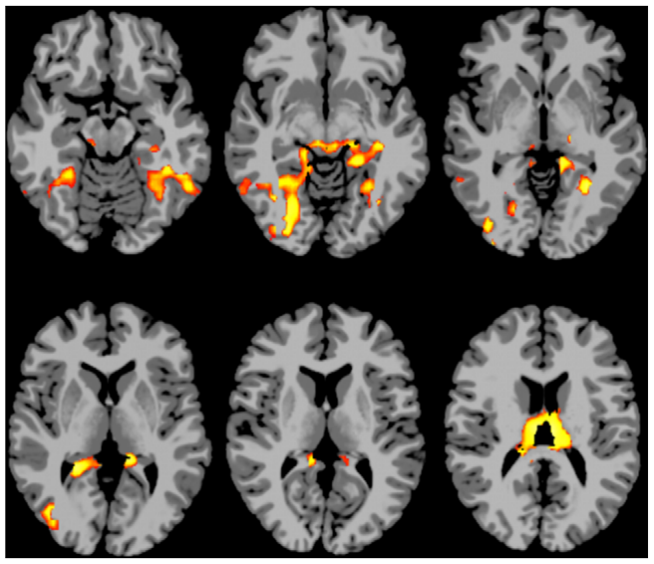
Areas showing increased connectivity to the medial temporal ROI with age. Slices are displayed using radiological convention (i.e., left is on the right).

The areas showing increases in connectivity to medial temporal cortex with age area shown in Figure 4. These include the fusiform gyrus and other visual regions, portions of the parahippocampal and retrosplenial cortices, and the dorsomedial nucleus of the thalamus, which has extensive connectivity to prefrontal regions [38].

**Figure 5:**
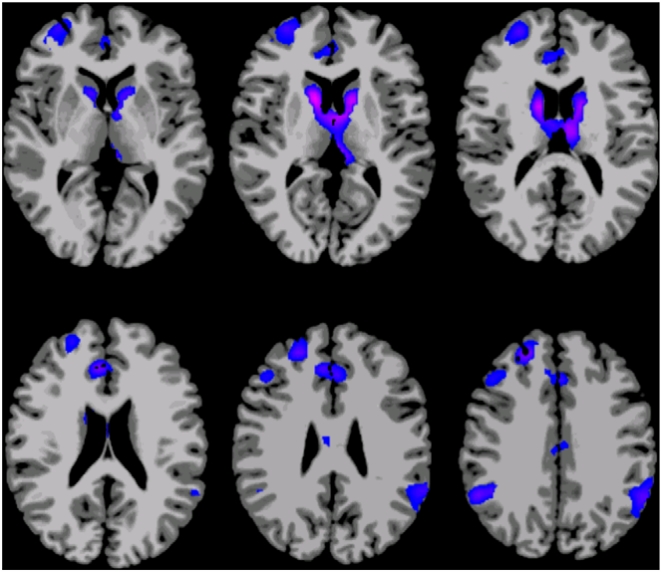
Areas showing decreased connectivity to the anterior cingulate cortex with age. Slices are displayed using radiological convention (i.e., left is on the right).

The areas showing decreased connectivity to the anterior cingulate cortex with age are shown in Figure 5. These include lateral parietal regions, right lateral frontal areas, the cerebellum, the caudate and the thalamus.

## Discussion

We have found that aging from young adulthood through middle age is associated with increased connectivity in paralimbic and subcortical areas, and decreased connectivity in a set of cortical regions including several nodes of the default mode network. These findings emerged both when we used the unweighted graph measure of degree and the weighted graph measure of vertex strength. Interestingly, a recent study using very different methods (that is, a multivariate analysis of conditional mutual information rather than a graph theory based analysis) to calculate the net connectivity in the resting brain also reported increasing connectivity with age in the orbitofrontal cortex and decreasing connectivity with age in the cingulate cortex [39]. Although the spatial extent of the findings in that study was limited to these two regions, this may have been related to differences in power arising from the different analyses used or the smaller sample size in the previous study.

### Increases in paralimbic cortex and subcortical areas

Increasing connectivity with age was found in limbic and paralimbic areas including lateral parts of the amygdala and hippocampus, the temporal pole, parahippocampal cortex, superior temporal sulcus, orbitofrontal cortex, and subcortical areas. Given that these regions are all involved in affective function, these connectivity changes may be related to changes in emotion processing with age.

There is a large literature documenting changes in emotion regulation capacity and emotional memory during mature aging. Many studies have reported that the capacity to regulate emotions increases with age from adolescence to late adulthood [1], [40], [41], [42]. Older adults tend to experience negative emotions less frequently and to control them better [43] and are less likely to attend to material with a negative emotional valence than to neutral or positive material [44]. In studies comparing memory for emotional material across different age groups, older adults remember less negative material, relative to neutral and positive material, than younger adults [45], [46], [47], [48]. In summary, older adults focus less on negative emotional information, show increased emotion regulation capacity, and have more positive overall emotional experiences than younger adults. These behavioral changes may be related to the age-related changes in connectivity reported here, particularly the increases in connectivity seen in limbic and paralimbic regions.

A secondary seed-based analysis examining age-related changes in connectivity to the medial temporal cortex revealed increased connectivity to the dorsomedial nucleus of the thalamus, which is highly connected to prefrontal regions [38] as well as increased connectivity to higher-level visual areas, including the fusiform gyrus, with increasing age. We speculate that these changes in limbic circuitry are related to known changes in emotion processing that occur with age. More specifically, we hypothesize that increased connectivity between medial temporal and frontal regions is related to increased emotion regulation ability, while increased connectivity between medial temporal and visual areas may enable older adults to selectively focus attention on information with a positive valence. Further studies are needed assessing various aspects of emotion regulation capacity and emotional memory and correlating these with connectivity measures to determine how the age-related connectivity patterns reported here relate to emotion processing.

### Decreases in default mode regions

A decrease in connectivity of default mode areas with age is consistent with prior literature reporting decreasing default mode connectivity in older adults that examined seed-region correlations or selected components from independent components analyses [7], [8], [49]. However, the findings reported here emerged from a whole-brain voxel-based analysis that in no way incorporated a-priori expectations regarding regions of interest in the default mode areas. Thus, they suggest that some of the most prominent decreases in network connectivity occurring throughout the brain involve the default mode network. Furthermore, as this study did not include elderly subjects, the findings indicate these changes are occurring in the early and middle stages of adulthood.

This study of changes in network properties during mature aging complements recent literature reporting changes in the whole-brain networks of children during development. Increases in long-range functional connections and decreases in short-range connections were found throughout the brain during development from childhood to young adulthood [50], [51], [52]. The default mode regions in particular were found to develop from a sparsely connected set of regions to a cohesive functional network [53]. In contrast, during mature aging and senescence, these brain areas apparently decrease their connectivity. The rise and fall of connectivity in this network over a lifetime thus coincides in a gross sense with the rise and fall of cognitive function.

Indeed, there is a growing literature suggesting that integrity of the network connections associated with default mode regions is critical to cognitive function [8], [26], [49], [54]. Thus decreasing connectivity in these regions may be responsible for age-related cognitive decline. Studies examining the relationship between specific cognitive abilities and network properties using measures such as those shown here provide a promising new approach for probing brain-behaviour relationships. Along these lines, a recent study relating network properties in structural brain networks of elderly subjects to cognitive measures reported, among other findings, that executive function was most correlated with regional efficiency in the posterior cingulate cortex [55]. Extending such work to voxel-wise analyses could provide a more detailed view of the neurobiological basis for cognitive decline.

A secondary seed based analysis, examining changes in connectivity to the anterior cingulate cortex with age, revealed decreased connectivity between this area and lateral parietal components of the default mode network, as well as decreased connectivity to lateral frontal areas, the caudate nucleus, and the dorsomedial portion of the thalamus with age. More work is needed to determine how this decrease in connectivity to other highly connected, associative brain areas is related to cognitive function.

### Effects of head motion on findings

There was a relationship between frame-to-frame head motion and age that was approaching significance in our data. Given this potential confound, we controlled for motion both by including head motion as a covariate in the group-level analysis, and by analyzing a subset of the data in which head motion was unrelated to age. The approach of covarying motion at the group level had very little effect on the results. Examining a subset of the data had more substantial effects, suggesting nonlinear effects of head motion on functional connectivity that cannot be controlled for by including motion as a regressor in a linear model. This has implications for other studies, in that it suggests that regressing out head motion in the group level analysis may not be an effective approach for controlling for this confound. Fortunately, we had sufficient data in each subject to allow us to analyze a subset of data that did not have any relationship between head motion and age. The findings were qualitatively similar to the full data set, showing increased degree of connectivity with age in limbic areas and paralimbic cortices and decreases in degree of connectivity in default mode regions. However, there were some differences.

One interesting difference between results from the full data set and results from the motion-balanced data set was seen in the posterior cingulate cortex. This region was prominent in our original map of age-related changes in connectivity, but disappeared when we controlled for head motion. This suggests that the connectivity in the posterior cingulate may be more related to head motion that to age. This is interesting given that two recent papers describing the confounding effects of head motion on functional connectivity patterns both reported that head motion was related to connectivity estimates in the posterior cingulate cortex [36], [37]. Further work is needed to determine if this is simply related to topology of the region and how that makes it susceptible to movement artifact, or if there is some relationship between functional connectivity of this region and the tendency of subjects to hold their heads still.

### Study limitations

Although the age-related connectivity patterns found in this study are consistent with the known effects of increased emotion regulation and decreased cognitive function with age, we did not collect behavioral data on these subjects. Therefore, we cannot be sure that the changes in connectivity found are really related to these aspects of mental function. Furthermore, the cross-sectional nature of this study limits data interpretation. Future studies that collect both behavioral and imaging data on the same subjects in a longitudinal fashion are recommended.

In addition, it should be kept in mind that large sample-size studies can identify small effects. In terms of human brain imaging, this is a fairly large study, and the age-related brain patterns reported may therefore correspond to effects that are small relative to those reported in other imaging studies.

### Conclusion

An emerging method for assessing network properties in the brain at the voxel level allowed identification of those regions of the brain that exhibit significant changes in the degree of connectivity during healthy aging. Increases in connectivity in paralimbic and subcortical regions and decreases in connectivity in default mode areas were found, consistent with the literature on increasing emotion regulation capacity and decreasing cognitive function with age. This approach provides a promising new tool for investigating the functional architecture of the human brain and its relationship to cognitive, clinical, and demographic variables.

## Supporting Information

Figure S1 **Map showing brain areas where age is correlated with degree of connectivity in the 59 subjects scanned on the Trio A scanner.** Results are displayed at an uncorrected p<0.05 level. Red/yellow areas indicate regions where connectivity increases with age, blue/purple areas indicate regions where connectivity decreases with age. Slices are shown using radiological convention (i.e. left is on the right). Although power is reduced due to the decrease in sample size, the qualitative pattern is similar to that seen in the full group of subjects.
(TIF)

Figure S2 **Map showing brain areas where there is a main effect of age on vertex strength, displayed at a whole brain corrected p<0.05 level.** Red/yellow areas indicate regions where connectivity increases with age, blue/purple areas indicate regions where connectivity decreases with age. Slices are shown using radiological convention (i.e. left is on the right).
(TIF)

Figure S3 **Map showing brain areas where there is a main effect of age on degree of connectivity as computed in a model that incorporated frame-to-frame head motion as a regressor.** Results displayed at a whole brain corrected p<0.05 level. Red/yellow areas indicate regions where connectivity increases with age, blue/purple areas indicate regions where connectivity decreases with age. Slices are shown using radiological convention (i.e. left is on the right). The inclusion of the motion regressor had little effect, as evidenced by similarity to Figure 2.
(TIF)

## References

[1] Gross JJ, Carstensen LL, Pasupathi M, Tsai J, Skorpen CG, et al (1997) Emotion and aging: experience, expression, and control. Psychol Aging 12: 590–599. 9416628 10.1037//0882-7974.12.4.590

[2] Baltes PB, Lindenberger U (1997) Emergence of a powerful connection between sensory and cognitive functions across the adult life span: a new window to the study of cognitive aging? Psychol Aging 12: 12–21. 9100264 10.1037//0882-7974.12.1.12

[3] Park DC, Lautenschlager G, Hedden T, Davidson NS, Smith AD, et al (2002) Models of visuospatial and verbal memory across the adult life span. Psychol Aging 17: 299–320. 12061414

[4] Park DC, Smith AD, Lautenschlager G, Earles JL, Frieske D, et al (1996) Mediators of long-term memory performance across the life span. Psychol Aging 11: 621–637. 9000294 10.1037//0882-7974.11.4.621

[5] Salthouse TA, Ferrer-Caja E (2003) What needs to be explained to account for age-related effects on multiple cognitive variables? Psychol Aging 18: 91–110. 12641315 10.1037/0882-7974.18.1.91

[6] Achard S, Bullmore E (2007) Efficiency and cost of economical brain functional networks. PLoS Comput Biol 3: e17. 17274684 10.1371/journal.pcbi.0030017 PMC1794324

[7] Andrews-Hanna JR, Snyder AZ, Vincent JL, Lustig C, Head D, et al (2007) Disruption of large-scale brain systems in advanced aging. Neuron 56: 924–935. 18054866 10.1016/j.neuron.2007.10.038 PMC2709284

[8] Damoiseaux JS, Beckmann CF, Arigita EJ, Barkhof F, Scheltens P, et al (2008) Reduced resting-state brain activity in the “default network” in normal aging. Cereb Cortex 18: 1856–1864. 18063564 10.1093/cercor/bhm207

[9] Grady CL, Springer MV, Hongwanishkul D, McIntosh AR, Winocur G (2006) Age-related changes in brain activity across the adult lifespan. J Cogn Neurosci 18: 227–241. 16494683 10.1162/089892906775783705

[10] Hedden T, Gabrieli JD (2004) Insights into the ageing mind: a view from cognitive neuroscience. Nat Rev Neurosci 5: 87–96. 14735112 10.1038/nrn1323

[11] Raz N, Gunning-Dixon F, Head D, Rodrigue KM, Williamson A, et al (2004) Aging, sexual dimorphism, and hemispheric asymmetry of the cerebral cortex: replicability of regional differences in volume. Neurobiology of aging 25: 377–396. 15123343 10.1016/S0197-4580(03)00118-0

[12] Bentourkia M, Bol A, Ivanoiu A, Labar D, Sibomana M, et al (2000) Comparison of regional cerebral blood flow and glucose metabolism in the normal brain: effect of aging. Journal of the neurological sciences 181: 19–28. 11099707 10.1016/s0022-510x(00)00396-8

[13] Cabeza R, Daselaar SM, Dolcos F, Prince SE, Budde M, et al (2004) Task-independent and task-specific age effects on brain activity during working memory, visual attention and episodic retrieval. Cerebral cortex 14: 364–375. 15028641 10.1093/cercor/bhg133

[14] Grady CL, Maisog JM, Horwitz B, Ungerleider LG, Mentis MJ, et al (1994) Age-related changes in cortical blood flow activation during visual processing of faces and location. The Journal of neuroscience : the official journal of the Society for Neuroscience 14: 1450–1462. 8126548 10.1523/JNEUROSCI.14-03-01450.1994 PMC6577560

[15] Good CD, Johnsrude IS, Ashburner J, Henson RN, Friston KJ, et al (2001) A voxel-based morphometric study of ageing in 465 normal adult human brains. Neuroimage 14: 21–36. 11525331 10.1006/nimg.2001.0786

[16] Bullmore E, Sporns O (2009) Complex brain networks: graph theoretical analysis of structural and functional systems. Nat Rev Neurosci 10: 186–198. 19190637 10.1038/nrn2575

[17] Gong G, Rosa-Neto P, Carbonell F, Chen ZJ, He Y, et al (2009) Age- and gender-related differences in the cortical anatomical network. J Neurosci 29: 15684–15693. 20016083 10.1523/JNEUROSCI.2308-09.2009 PMC2831804

[18] Meunier D, Achard S, Morcom A, Bullmore E (2009) Age-related changes in modular organization of human brain functional networks. Neuroimage 44: 715–723. 19027073 10.1016/j.neuroimage.2008.09.062

[19] Shen X, Papademetris X, Constable RT (2010) Graph-theory based parcellation of functional subunits in the brain from resting-state fMRI data. Neuroimage 50: 1027–1035. 20060479 10.1016/j.neuroimage.2009.12.119 PMC3062848

[20] Tzourio-Mazoyer N, Landeau B, Papathanassiou D, Crivello F, Etard O, et al (2002) Automated anatomical labeling of activations in SPM using a macroscopic anatomical parcellation of the MNI MRI single-subject brain. Neuroimage 15: 273–289. 11771995 10.1006/nimg.2001.0978

[21] Song M, Liu Y, Zhou Y, Wang K, Yu C, et al (2009) Default network and intelligence difference. Conf Proc IEEE Eng Med Biol Soc 2009: 2212–2215. 19964951 10.1109/IEMBS.2009.5334874

[22] van den Heuvel MP, Stam CJ, Boersma M, Hulshoff Pol HE (2008) Small-world and scale-free organization of voxel-based resting-state functional connectivity in the human brain. Neuroimage 43: 528–539. 18786642 10.1016/j.neuroimage.2008.08.010

[23] Buckner RL, Sepulcre J, Talukdar T, Krienen FM, Liu H, et al (2009) Cortical hubs revealed by intrinsic functional connectivity: mapping, assessment of stability, and relation to Alzheimer's disease. J Neurosci 29: 1860–1873. 19211893 10.1523/JNEUROSCI.5062-08.2009 PMC2750039

[24] Cole MW, Pathak S, Schneider W (2010) Identifying the brain's most globally connected regions. Neuroimage 49: 3132–3148. 19909818 10.1016/j.neuroimage.2009.11.001

[25] Tomasi D, Volkow ND (2011) Functional connectivity hubs in the human brain. Neuroimage 10.1016/j.neuroimage.2011.05.024 PMC3129362 21609769

[26] van den Heuvel MP, Stam CJ, Kahn RS, Hulshoff Pol HE (2009) Efficiency of functional brain networks and intellectual performance. J Neurosci 29: 7619–7624. 19515930 10.1523/JNEUROSCI.1443-09.2009 PMC6665421

[27] Burdette JH, Laurienti PJ, Espeland MA, Morgan A, Telesford Q, et al (2010) Using network science to evaluate exercise-associated brain changes in older adults. Front Aging Neurosci 2: 23. 20589103 10.3389/fnagi.2010.00023 PMC2893375

[28] Martuzzi R, Ramani R, Qiu M, Shen X, Papademetris X, et al (2011) A whole-brain voxel based measure of intrinsic connectivity contrast reveals local changes in tissue connectivity with anesthetic without a priori assumptions on thresholds or regions of interest. Neuroimage 10.1016/j.neuroimage.2011.06.075 PMC3183817 21763437

[29] Achard S, Salvador R, Whitcher B, Suckling J, Bullmore E (2006) A resilient, low-frequency, small-world human brain functional network with highly connected association cortical hubs. J Neurosci 26: 63–72. 16399673 10.1523/JNEUROSCI.3874-05.2006 PMC6674299

[30] Barrat A, Barthelemy M, Pastor-Satorras R, Vespignani A (2004) The architecture of complex weighted networks. Proc Natl Acad Sci U S A 101: 3747–3752. 15007165 10.1073/pnas.0400087101 PMC374315

[31] Martuzzi R, Ramani R, Qiu M, Rajeevan N, Constable RT (2010) Functional connectivity and alterations in baseline brain state in humans. Neuroimage 49: 823–834. 19631277 10.1016/j.neuroimage.2009.07.028 PMC2764802

[32] Scheinost D, Benjamin J, Lacadie CM, Vohr B, Schneider KC, et al (2012) The Intrinsic Connectivity Distribution: A Novel Contrast Measure Reflecting Voxel Level Functional Connectivity. Neuro Image 10.1016/j.neuroimage.2012.05.073 PMC3538880 22659477

[33] Holmes CJ, Hoge R, Collins L, Woods R, Toga AW, et al (1998) Enhancement of MR images using registration for signal averaging. Journal of Computer Assisted Tomography 22: 324–333. 9530404 10.1097/00004728-199803000-00032

[34] Papademetris X, Jackowski AP, Schultz RT, Staib LH, Duncan JS (2004) Integrated intensity and point-feature nonrigid registration. Medical Image Computing and Computer-Assisted Intervention - Miccai 2004, Pt 1, Proceedings 3216: 763–770. 10.1901/jaba.2001.3216-763 PMC2869095 20473359

[35] Lacadie CM, Fulbright RK, Rajeevan N, Constable RT, Papademetris X (2008) More accurate Talairach coordinates for neuroimaging using non-linear registration. Neuro Image 42: 717–725. 18572418 10.1016/j.neuroimage.2008.04.240 PMC2603575

[36] Power JD, Barnes KA, Snyder AZ, Schlaggar BL, Petersen SE (2012) Spurious but systematic correlations in functional connectivity MRI networks arise from subject motion. Neuro Image 59: 2142–2154. 22019881 10.1016/j.neuroimage.2011.10.018 PMC3254728

[37] Van Dijk KR, Sabuncu MR, Buckner RL (2012) The influence of head motion on intrinsic functional connectivity MRI. Neuro Image 59: 431–438. 21810475 10.1016/j.neuroimage.2011.07.044 PMC3683830

[38] Jelsing J, Hay-Schmidt A, Dyrby T, Hemmingsen R, Uylings HB, et al (2006) The prefrontal cortex in the Gottingen minipig brain defined by neural projection criteria and cytoarchitecture. Brain Res Bull 70: 322–336. 17027768 10.1016/j.brainresbull.2006.06.009

[39] Salvador R, Anguera M, Gomar JJ, Bullmore ET, Pomarol-Clotet E (2010) Conditional mutual information maps as descriptors of net connectivity levels in the brain. Front Neuroinform 4: 115. 21151357 10.3389/fninf.2010.00115 PMC2995463

[40] Labouvie-Vief G, Hakim-Larson J, DeVoe M, Schoeberlein S (1989) Emotions and self-regulation: a life span view. Human Development 32: 279–299.

[41] Lawton MP, Kleban MH, Rajagopal D, Dean J (1992) Dimensions of affective experience in three age groups. Psychol Aging 7: 171–184. 1610505 10.1037//0882-7974.7.2.171

[42] Orgeta V (2009) Specificity of age differences in emotion regulation. Aging Ment Health 13: 818–826. 19888702 10.1080/13607860902989661

[43] Carstensen LL, Pasupathi M, Mayr U, Nesselroade JR (2000) Emotional experience in everyday life across the adult life span. J Pers Soc Psychol 79: 644–655. 11045744

[44] Mather M, Carstensen LL (2003) Aging and attentional biases for emotional faces. Psychol Sci 14: 409–415. 12930469 10.1111/1467-9280.01455

[45] Charles ST, Mather M, Carstensen LL (2003) Aging and emotional memory: the forgettable nature of negative images for older adults. J Exp Psychol Gen 132: 310–324. 12825643 10.1037/0096-3445.132.2.310

[46] Grady CL, Hongwanishkul D, Keightley M, Lee W, Hasher L (2007) The effect of age on memory for emotional faces. Neuropsychology 21: 371–380. 17484600 10.1037/0894-4105.21.3.371

[47] Levine LJ, Bluck S (1997) Experienced and remembered emotional intensity in older adults. Psychol Aging 12: 514–523. 9308098 10.1037//0882-7974.12.3.514

[48] Thomas RC, Hasher L (2006) The influence of emotional valence on age differences in early processing and memory. Psychol Aging 21: 821–825. 17201502 10.1037/0882-7974.21.4.821 PMC1764613

[49] Sambataro F, Murty VP, Callicott JH, Tan HY, Das S, et al (2010) Age-related alterations in default mode network: Impact on working memory performance. Neurobiol Aging 31: 839–852. 18674847 10.1016/j.neurobiolaging.2008.05.022 PMC2842461

[50] Fair DA, Cohen AL, Power JD, Dosenbach NU, Church JA, et al (2009) Functional brain networks develop from a “local to distributed” organization. PLoS Comput Biol 5: e1000381. 19412534 10.1371/journal.pcbi.1000381 PMC2671306

[51] Fair DA, Dosenbach NU, Church JA, Cohen AL, Brahmbhatt S, et al (2007) Development of distinct control networks through segregation and integration. Proc Natl Acad Sci U S A 104: 13507–13512. 17679691 10.1073/pnas.0705843104 PMC1940033

[52] Supekar K, Musen M, Menon V (2009) Development of large-scale functional brain networks in children. PLoS Biol 7: e1000157. 19621066 10.1371/journal.pbio.1000157 PMC2705656

[53] Fair DA, Cohen AL, Dosenbach NU, Church JA, Miezin FM, et al (2008) The maturing architecture of the brain's default network. Proc Natl Acad Sci U S A 105: 4028–4032. 18322013 10.1073/pnas.0800376105 PMC2268790

[54] Hampson M, Driesen NR, Skudlarski P, Gore JC, Constable RT (2006) Brain connectivity related to working memory performance. Journal of Neuroscience 26: 13338–13343. 17182784 10.1523/JNEUROSCI.3408-06.2006 PMC2677699

[55] Wen W, Zhu W, He Y, Kochan NA, Reppermund S, et al (2011) Discrete neuroanatomical networks are associated with specific cognitive abilities in old age. J Neurosci 31: 1204–1212. 21273405 10.1523/JNEUROSCI.4085-10.2011 PMC6623602

