# Supplementary figures and images for: Intrinsic Brain Connectivity Related to Age in Young and Middle Aged Adults
Source: PLoS One. 2012 Sep 11;7(9):e44067. doi: 10.1371/journal.pone.0044067 (PMC3439483; doi:10.1371/journal.pone.0044067)

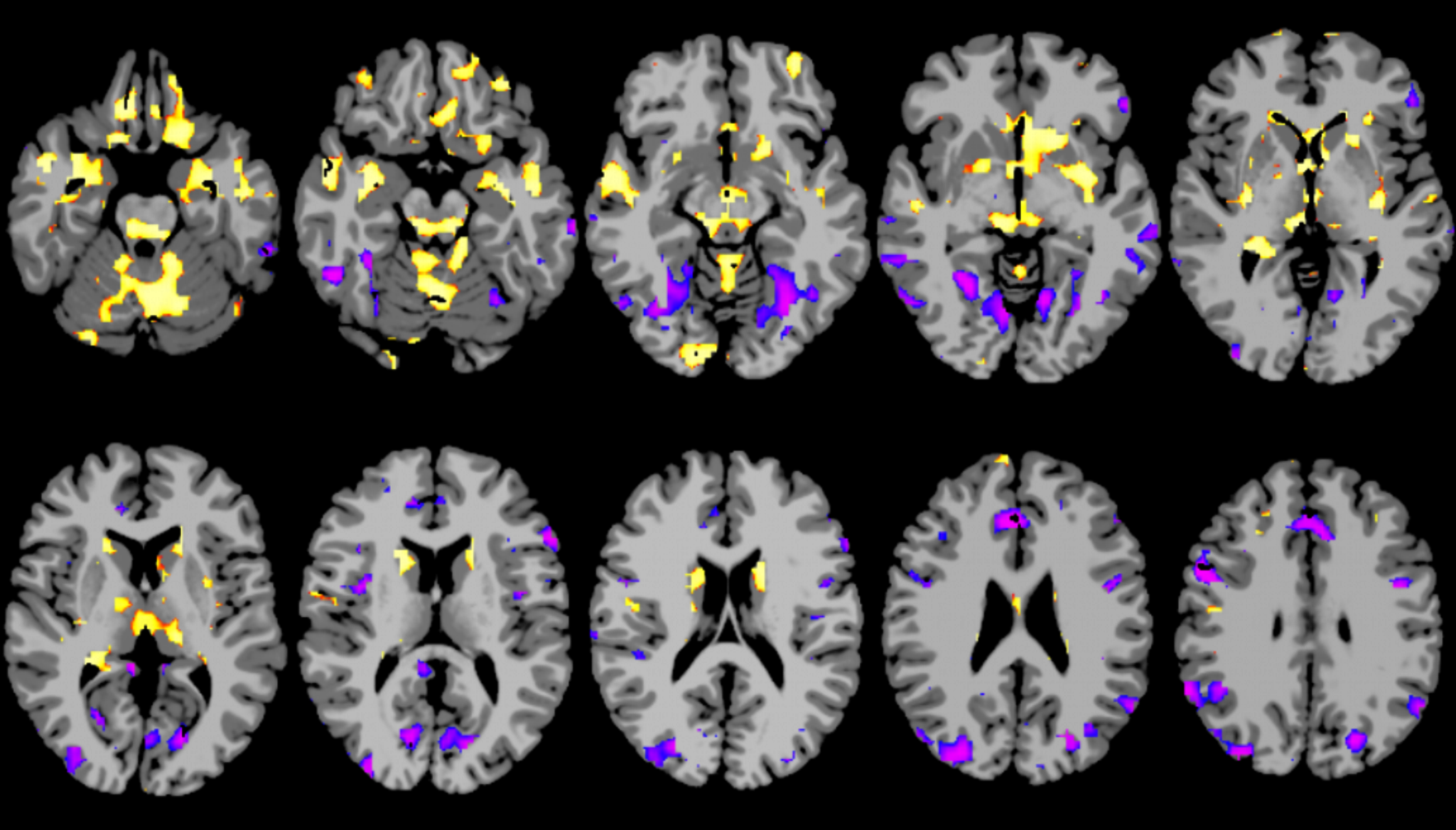

Supplement: Figure S1 — Map showing brain areas where age is correlated with degree of connectivity in the 59 subjects scanned on the Trio A scanner. Results are displayed at an uncorrected p<0.05 level. Red/yellow areas indicate regions where connectivity increases with age, blue/purple areas indicate regions where connectivity decreases with age. Slices are shown using radiological convention (i.e. left is on the right). Although power is reduced due to the decrease in sample size, the qualitative pattern is similar to that seen in the full group of subjects. (TIF) [file pone.0044067.s001.tif]

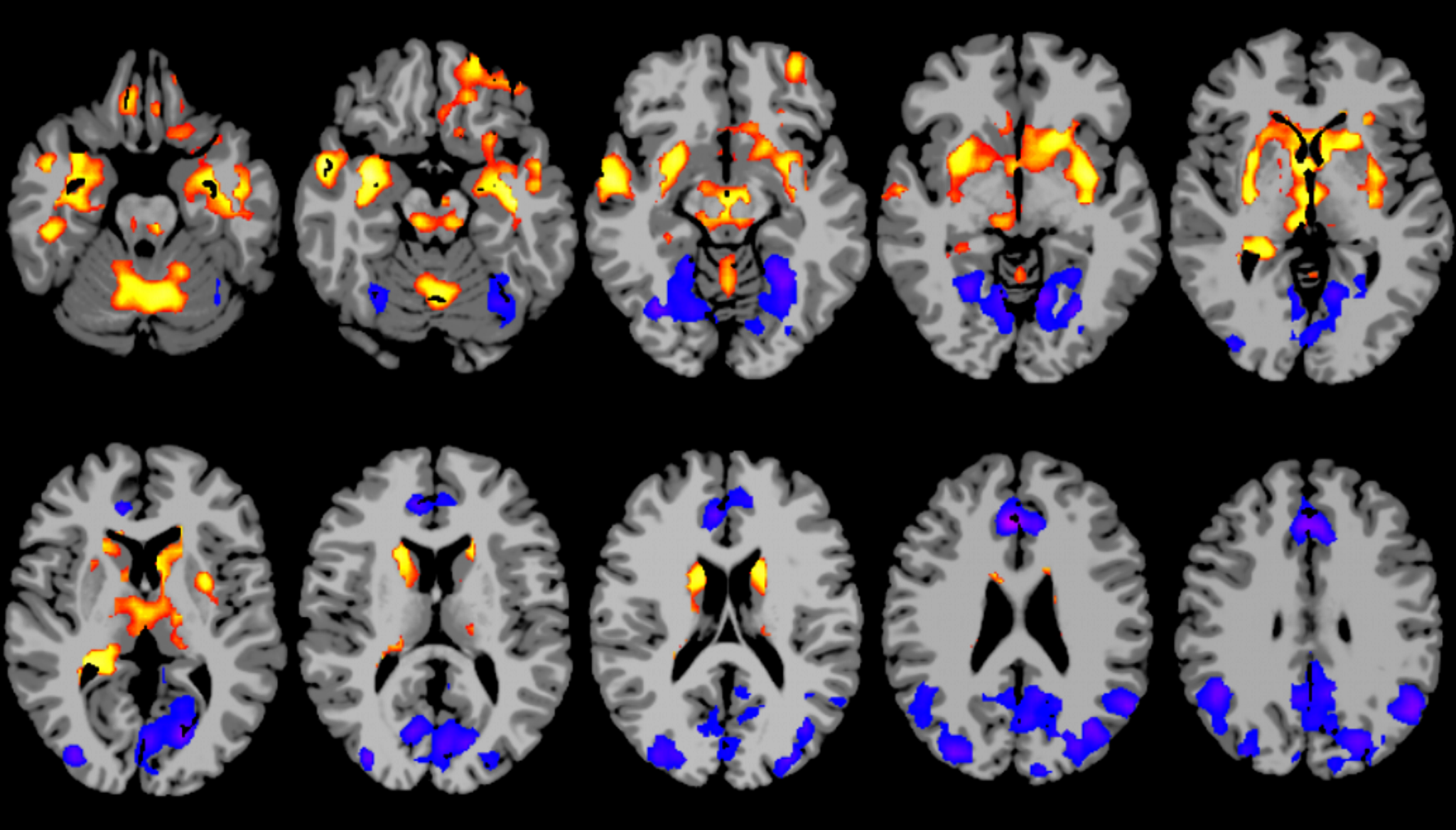

Supplement: Figure S2 — Map showing brain areas where there is a main effect of age on vertex strength, displayed at a whole brain corrected p<0.05 level. Red/yellow areas indicate regions where connectivity increases with age, blue/purple areas indicate regions where connectivity decreases with age. Slices are shown using radiological convention (i.e. left is on the right). (TIF) [file pone.0044067.s002.tif]

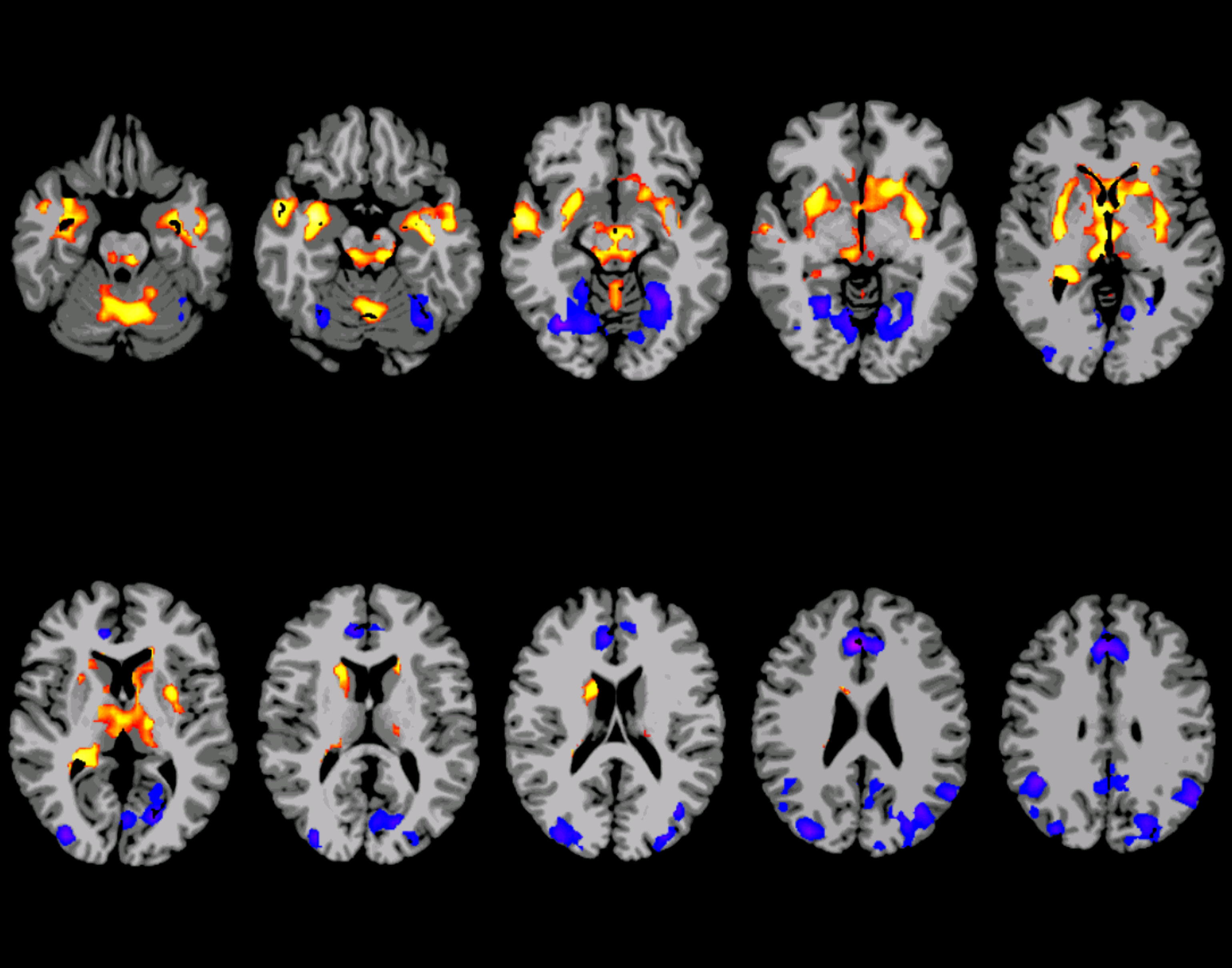

Supplement: Figure S3 — Map showing brain areas where there is a main effect of age on degree of connectivity as computed in a model that incorporated frame-to-frame head motion as a regressor. Results displayed at a whole brain corrected p<0.05 level. Red/yellow areas indicate regions where connectivity increases with age, blue/purple areas indicate regions where connectivity decreases with age. Slices are shown using radiological convention (i.e. left is on the right). The inclusion of the motion regressor had little effect, as evidenced by similarity to Figure 2. (TIF) [file pone.0044067.s003.tif]
